# A Hybrid 2D/3D Approach for Neural Differentiation Into Telencephalic Organoids and Efficient Modulation of FGF8 Signaling

**DOI:** 10.21769/BioProtoc.5354

**Published:** 2025-06-20

**Authors:** Michele Bertacchi, Gwendoline Maharaux, Michèle Studer

**Affiliations:** Institut de Biologie Valrose (iBV), University Côte d’Azur (UniCA), CNRS, Inserm, Nice, France

**Keywords:** Telencephalic organoids, Neural differentiation, Brain organoids, FGF8, Brain patterning, hiPSCs

## Abstract

Human brain development relies on a finely tuned balance between the proliferation and differentiation of neural progenitor cells, followed by the migration, differentiation, and connectivity of post-mitotic neurons with region-specific identities. These processes are orchestrated by gradients of morphogens, such as FGF8. Disruption of this developmental balance can lead to brain malformations, which underlie a range of complex neurodevelopmental disorders, including epilepsy, autism, and intellectual disabilities. Studying the early stages of human brain development, whether under normal or pathological conditions, remains challenging due to ethical and technical limitations inherent to working with human fetal tissue. Recently, human brain organoids have emerged as a powerful in vitro alternative, allowing researchers to model key aspects of early brain development while circumventing many of these constraints. Unlike traditional 2D cultures, where neural progenitors and neurons are grown on flat surfaces, 3D organoids form floating self-organizing aggregates that better replicate the cellular diversity and tissue architecture of the developing brain. However, 3D organoid protocols often suffer from significant variability between batches and individual organoids. Furthermore, few existing protocols directly manipulate key morphogen signaling pathways or provide detailed analyses of the resulting effects on regional brain patterning.

• To address these limitations, we developed a hybrid 2D/3D approach for the rapid and efficient induction of telencephalic organoids that recapitulate major steps of anterior brain development. Starting from human induced pluripotent stem cells (hiPSCs), our protocol begins with 2D neural induction using small-molecule inhibitors to achieve fast and homogenous production of neural progenitors (NPs). After dissociation, NPs are reaggregated in Matrigel droplets and cultured in spinning mini-bioreactors, where they self-organize into neural rosettes and neuroepithelial structures, surrounded by differentiating neurons. Activation of the FGF signaling pathway through the controlled addition of FGF8 to the culture medium will modulate regional identity within developing organoids, leading to the formation of distinct co-developing domains within a single organoid. Our protocol combines the speed and reproducibility of 2D induction with the structural and cellular complexity of 3D telencephalic organoids. The ability to manipulate signaling pathways provides an additional opportunity to further increase system complexity, enabling the simultaneous development of multiple distinct brain regions within a single organoid. This versatile system facilitates the study of key cellular and molecular mechanisms driving early human brain development across both telencephalic and non-telencephalic areas.

Key features

• This protocol builds on the method established by Chambers et al. [1] for generating 2D neural progenitors, followed by dissociation and reaggregation into 3D brain organoids.

• For optimal growth and maturation, telencephalic organoids are cultured in spinning mini-bioreactors [2] or on orbital shakers.

• The protocol enables the generation of telencephalic neural progenitors in 10 days and produces 3D telencephalic organoids containing neocortical neurons within one month of culture.

• Addition of morphogens in the culture medium (example: FGF8) enhances cellular heterogeneity, promoting the emergence of distinct brain domains within a single organoid.

## Graphical overview


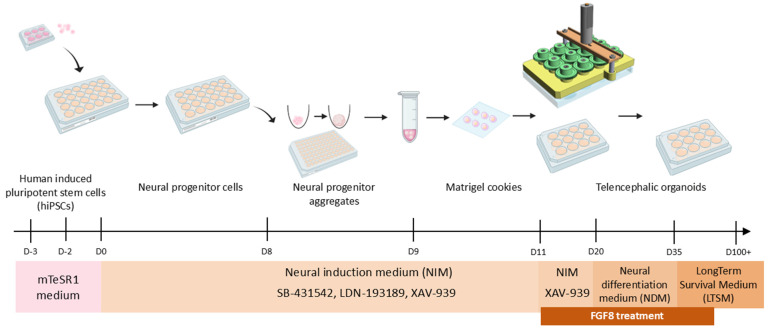


**Schematic of the hybrid 2D/3D protocol for fast and reproducible generation of human telencephalic organoids.** 3D model of SpinΩ mini-bioreactor is shown as in Qian et al. [2], while other images were obtained from BioRender (https://www.biorender.com). A copy of this image is available in the BioRender template database (link: https://app.biorender.com/profile/template/details/t-684295eb6d4d1817efba72c3-schematic-of-the-hybrid-2d3d-protocol-for-generation-of-huma).

## Background

Self-organizing human brain organoids offer an unprecedented tool for modeling early neural development in vitro [3–5]. They provide an ethical and practical alternative for studying both normal and pathological processes during early human fetal brain development.

Different approaches have been developed to induce neural tissue in vitro, each with its own advantages and limitations. Traditional 2D neural induction protocols involve culturing cells on a bidimensional surface [1,6,7]. These methods are fast and reproducible, allowing for the efficient generation of uniform neural progenitors (NPs), but they can be limited in terms of the formation of complex tissue architecture and cellular heterogeneity. In contrast, more recent adaptations of neural induction protocols in 3D generate brain organoids, aggregates of NPs and neurons that self-organize into three-dimensional structures mimicking early fetal brain architecture [8,9]. These organoids develop neuroepithelial structures with ventricle-like cavities, surrounded by neurons arranged in a cortical plate-like organization [10]. This enhanced spatial organization is accompanied by increased cellular diversity and heterogeneity [11], better reflecting the variety of progenitors and neuronal subtypes found in the in vivo telencephalon. However, this increased complexity introduces greater inter-organoid variability [11,12], especially in unguided differentiation protocols that rely solely on intrinsic self-organization.

Building upon previous methods [1,2,13], we have developed a hybrid approach that combines the speed and homogeneity of 2D neural induction with the structural complexity of 3D organoid culture to create a more reproducible cerebral organoid protocol. The use of SpinΩ mini-rotors [13] or orbital shakers, coupled with inclusion in Matrigel droplets to provide an external scaffold [14], supports optimal tissue growth and self-organization, resulting in organoids that recapitulate key aspects of fetal cortical development.

Previous studies have shown that pluripotent cells preferentially adopt a dorsal/anterior (i.e., telencephalic) neural fate when shielded from posteriorizing factors such as TGFβ, BMPs, retinoic acid, and WNTs [15]. To promote efficient acquisition of an anterior telencephalic fate, our protocol employs a combination of chemical inhibitors that suppress the endogenous production and action of TGFβ, BMP, and WNT factors. To further modulate anterior identity, we incorporate extrinsic morphogens such as FGF8 in a dose- and time-dependent manner [16]. Specifically, FGF8 acts as a regulator of anterior-posterior (A/P) and dorsal-ventral (D/V) identities in forebrain cells, instructing the development of multiple regional domains. This includes the dorsal and ventral telencephalon, as well as diencephalic and mesencephalic regions that co-develop within the same organoid while maintaining spatial segregation [16].

In summary, our optimized 2D/3D hybrid culture system enables the rapid and consistent generation of telencephalic FOXG1+ tissue and provides a robust platform to assess the effects of FGF8 and other morphogens on early neural differentiation and regional identity. This protocol offers a biologically accurate and reproducible cell culture approach that serves as a valuable tool for advancing our understanding of human brain development and associated neurological diseases.

## Materials and reagents

**Biological materials**

1. Human induced pluripotent stem cells (hiPSCs), HMGU1 line, kind gift of Dr. Drukker. MTA approval obtained from the Helmholtz Zentrum München

**Important:** When using other hiPSC lines, please be aware that the concentration of chemical compounds and the duration of various steps may require adjustment. We recently achieved good results with PGP1 hiPSCs by implementing only minor modifications to the protocol.

**Reagents**

1. mTeSR1 Medium kit (STEMCELL Technologies, catalog number: 85850)

2. mTeSR Plus Medium kit (STEMCELL Technologies, catalog number: 100-0276)

3. Matrigel matrix (Matrigel) (Corning, catalog number: 354234)

4. DMEM-F12 medium (DMEM-F12) (Thermo Fisher Scientific, catalog number: 31331028)

5. Versene (Thermo Fisher Scientific, catalog number: 15040066)

6. Dulbecco’s phosphate buffered saline (DPBS or PBS) 1× (Thermo Fisher Scientific, catalog number: 14190169)

7. Neurobasal medium (Thermo Fisher Scientific, catalog number: 21103049)

8. Accutase solution (Sigma-Aldrich, catalog number: A6964)

9. Dihydrochloride ROCK inhibitor Y-27632 (ROCKi) (MedChemExpress, catalog number: HY-10583, or STEMCELL Technologies, catalog number: 72304; prepare a 10 mM stock solution in DMSO; store aliquots at -20 °C for up to 6 months)

10. N-2 supplement (100×) (N2) (Thermo Fisher Scientific, catalog number: 17502-048)

11. B-27 supplement minus vitamin A (50×) (Thermo Fisher Scientific, catalog number: 12587-010)

12. GlutaMAX (Thermo Fisher Scientific, catalog number: 35050038)

13. MEM non-essential amino acids (NEAA) (Thermo Fisher Scientific, catalog number: 11140-035)

14. Sodium pyruvate (NaPyr) (Thermo Fisher Scientific, catalog number: 11360070)

15. Beta-mercaptoethanol (Thermo Fisher Scientific, catalog number: 31350-010)

16. Heparin (Sigma-Aldrich, catalog number: H3149)

17. Antibiotic/antimycotic solution (100×) (Anti/Anti) (Sigma-Aldrich, catalog number: A5955)

18. Penicillin-streptomycin (Pen/Strep) (5,000 U/mL) (Thermo Fisher Scientific, Gibco, catalog number: 15070063)

19. Dimethylsulfoxide (DMSO) (Sigma-Aldrich, catalog number: D4540-100ML)

20. LDN-193189 (LDN) (Sigma-Aldrich, catalog number: SML0559-5MG; prepare a 5 mM stock solution by adding 2.46 mL of DMSO to 5 mg of LDN; store aliquots at -20 °C for up to 6 months or at -80 °C for long-term)

21. SB-431542 (SB) (Sigma-Aldrich, catalog number: S4317-5MG; prepare a 10 mM stock solution by adding 1.3 mL of DMSO to 5 mg of SB; store aliquots at -20 °C for up to 6 months)

22. XAV-939 (XAV) (STEMCELL Technologies, catalog number: 72674; prepare a 10 mM stock solution by resuspending in DMSO; store aliquots at -20 °C for up to 6 months)

23. IWR-1-endo (IWR) (Sigma-Aldrich, catalog number: I0161-5MG; prepare a 3 mM stock solution by adding 4.065 mL of DMSO to 5 mg of IWR; store aliquots at -20 °C for up to 6 months)

24. Fetal bovine serum (FBS) (Thermo Fisher Scientific, catalog number: 10270-106)

25. BDNF (PeproTech, catalog number: 450-02; prepare a 50 ng/μL stock solution by resuspending in 1× PBS supplemented with 0.1% BSA; store aliquots at -20 °C for up to 6 months)

26. Recombinant human/mouse FGF-8b protein (FGF8) (R&D Systems, catalog number: 423-F8-025/CF; prepare a 25 ng/μL stock solution by resuspending in 1× PBS supplemented with 0.1% BSA; store aliquots at -20 °C for up to 6 months)

27. Bovine serum albumin (BSA) (IgG-free, protease-free) (Jackson ImmunoResearch, catalog number: 001-000-161; resuspend in 1× PBS at a concentration of 2%, filter, then aliquot and store at -20 °C)

28. Insulin (Sigma-Aldrich, catalog number: I9278-5ML)

29. 96% Ethanol (VWR Chemicals, catalog number: 83804.360 or similar)

**Solutions**

1. Matrigel coating solution (see Recipes)

2. mTeSR1 complete medium (see Recipes)

3. Neural induction medium (NIM) (see Recipes)

4. NIM with chemical inhibitors (see Recipes)

5. Neural differentiation medium (NDM) (see Recipes)

6. Long-term pro-survival medium (LTSM) (see Recipes)

**Recipes**

**1. Matrigel coating solution**

It is important to keep all components at 2–4 °C during the preparation of the Matrigel coating solution to prevent premature solidification. To ensure this, thaw the Matrigel aliquot and work on ice, keeping both the Matrigel and the DMEM/F-12 medium on ice while preparing solution 1. The volume of solution to prepare should be adjusted based on the number and size of the wells to be coated. Always prepare a fresh solution for each experiment and discard any leftover solution.

**Table:** 

Reagent	Final concentration	Volume
DMEM/F-12	99.5%	9.95 mL
Matrigel	0.5%	50 μL
Total	100%	10 mL

**2. mTeSR1 complete medium**

Thaw the frozen mTeSR1 medium supplement from the mTeSR1 Medium kit at 4 °C overnight, as indicated in the manufacturer's instructions. Once thawed, gently mix the supplement by inverting the bottle a few times, then aseptically transfer all contents of the mTeSR1 supplement bottle into the mTeSR1 basal medium. Swirl the bottle gently to mix. The complete mTeSR1 medium can be stored at 4 °C for up to two weeks. Alternatively, for smaller-scale experiments, aliquot the mTeSR1 supplement into Falcon tubes and store at -20 °C. Thaw only the volume needed and dilute it with mTeSR1 basal medium (see table below for a 50 mL preparation example).

**Table:** 

Reagent	Final concentration	Volume
mTeSR 1 basal medium	80%	40 mL
mTeSR 1 5× supplement	20%	10 mL
Total	100%	50 mL

**3. Neural induction medium (NIM): d0-d20**

First, thaw the N-2 supplement and B27 minus vitamin A at room temperature. Then, prepare 50 mL of NIM solution and filter it at the end of the preparation. The NIM solution can be stored at 4 °C for up to two weeks.

**Table:** 

Reagent	Final concentration	Volume
DMEM/F-12 with GlutaMAX	~92%	~46 mL
N-2 supplement (100×)	1×	500 μL
B-27 minus vitamin A (50×)	1×	1 mL
GlutaMAX (100×)	1×	500 μL
NEAA (100×)	1×	500 μL
NaPyr (100×)	1×	500 μL
Anti/Anti (100×)	0.5×	250 μL
Beta-mercaptoethanol (50 mM)	50 μM	50 μL
Heparin 10 mg/mL	2 μg/mL	10 μL
Total		50 mL

**4. NIM with chemical inhibitors: d0-d7**

Prepare 50 mL of NIM and sterilize it by filtration (see Recipe 3). To prepare complete NIM with chemical inhibitors, aliquot the required volume for a few days of use and freshly add SB-431542, LDN-193189, and XAV-939 (the table below provides an example preparation for 10 mL). While the TGF-β inhibitor SB-431542 and the BMP inhibitor LDN-193189 promote neural induction, the WNT inhibitor XAV-939 is used to promote anteriorization (telencephalic induction). The complete NIM solution can be stored at 4 °C for up to one week, protected from light. As an alternative to XAV-939, we also successfully used IWR-1-endo as a WNT inhibitor at a concentration of 3 μM. Both compounds were found to be functionally equivalent in our experiments.

**Table:** 

Reagent	Final concentration	Volume
LDN-193189 (5 mM)	0.25 μM	0.5 μL
SB-431542 (10 mM)	5 μM	5 μL
XAV-939 (10 mM)	2 μM	2 μL
NIM filtered medium	100%	10 mL

**5. Neural differentiation medium (NDM): d20-d25**

First, thaw N-2 supplement at room temperature. Then, prepare 50 mL of NDM solution and filter the solution at the end. The NDM solution can be stored at 4 °C for up to two weeks.

**Table:** 

Reagent	Final concentration	Volume
DMEM/F-12 with GlutaMAX	~46.2%	~23 mL
Neurobasal medium	~46.2%	~23 mL
N-2 supplement (100×)	1×	500 μL
GlutaMAX (100×)	1×	500 μL
NEAA (100×)	1×	500 μL
NaPyr (100×)	1×	500 μL
Anti/Anti (100×)	0.5×	250 μL
Beta-mercaptoethanol (50 mM)	50 μM	50 μL
Heparin 10 mg/mL	2 μg/mL	10 μL
Insulin	1×	12.5 μL
Total		50 mL

**6. Long-term pro-survival medium (LTSM): starting from d25 through the remainder of the culture period**

First, thaw the N-2 supplement and B27 minus vitamin A at room temperature, and thaw the Matrigel matrix at 4 °C or on ice. Next, prepare approximately 50 mL of LTSM solution and filter it before adding BDNF and the Matrigel matrix. The LTSM solution can be stored at 4 °C for up to two weeks. To prevent Matrigel precipitation, do not prewarm the solution to 37 °C before adding it to organoids; instead, prewarm it at room temperature for 15 min only.

**Table:** 

Reagent	Final concentration	Volume
Neurobasal medium	~90%	~45 mL
eN-2 supplement (100×)	1×	500 μL
B-27 minus vitamin A (50×)	1×	1 mL
GlutaMAX (100×)	1×	500 μL
NEAA (100×)	1×	500 μL
NaPyr (100×)	1×	500 μL
Anti/Anti (100×)	0.5×	250 μL
Beta-mercaptoethanol (50 mM)	50 μM	50 μL
Heparin (10 mg/mL)	2 μg/mL	10 μL
FBS	1%	500 μL
Filter		
Matrigel matrix	0.1%	50 μL
BDNF (50 ng/μL)	10 ng/mL	10 μL
Total		50 mL

**Laboratory supplies**

1. 6-well cell culture plates (Corning, Falcon, catalog number: 353224 or equivalent)

2. 12-well cell culture plates (Corning, Falcon, catalog number: 353225 or equivalent)

3. 24-well cell culture plates (Corning, Falcon, catalog number: 353226 or equivalent)

4. 96-well U-bottom ultra-low attachment plates (Corning, catalog number: CLS7007-24EA or Sarstedt, catalog number: 833925500)

5. 50 mL Falcon conical tubes (Corning, Falcon, catalog number: 352070 or similar)

6. 15 mL Falcon conical tubes (Corning, Falcon, catalog number: 352096 or similar)

7. 0.22 μm syringe filters (Sigma, catalog number: SLGP033RS or similar)

8. 5 mL serological pipettes (Corning, Falcon, catalog number: 357543 or similar)

9. 10 mL serological pipettes (Corning, Falcon, catalog number: 357551 or similar)

10. 25 mL serological pipettes (Corning, Falcon, catalog number: 357525 or similar)

11. Sterile PES 50 mL syringes (Fisher Scientific, catalog number: 14-955-455 or similar)

12. 10 μL pipette tips (Greiner Bio-one, catalog number: 771352 or equivalent)

13. 200 μL pipette tips (Greiner Bio-one, catalog number: 775352 or equivalent)

14. 1,000 μL pipette tips (Greiner Bio-one, catalog number: 777352 or equivalent)

15. 100 mm Petri dishes (Sarstedt, catalog number: 83.3902 or equivalent)

16. Parafilm (Heathrow Scientific, catalog number: HS234526A or equivalent)

17. 1.5 mL or 2 mL Eppendorf tubes (Dutscher, Eppendorf, catalog number: 033511 or similar)

## Equipment

1. Biological safety cabinet

2. Incubator 5% CO_2_ (Panasonic, model: KM-CC17R2E or similar)

3. SpinΩ mini-rotors (3Dnamics, model: 3DNA01). Alternatively, CO_2_-resistant orbital shaker (Fisher Scientific, catalog number: 88881102) and accessories (aluminum and rubber platforms; Fisher Scientific, catalog numbers: 88881122 and 88881123)

5. Laboratory centrifuge with rotors for 15 and 50 mL conical tubes (Eppendorf, model: 5810 R or similar)

6. Counting chambers, Bürker pattern (BlauBrand, catalog number: 718920 or similar)

7. Water bath

8. Pipetboy

9. Racks

10. Liquid nitrogen tank

11. Freezer (-20 °C) and refrigerator (2–8 °C)

## Procedure

**A. hiPSC culture for maintenance and amplification**

1. Prepare the coating of a suitable plate (typically one well of a 6-well plate) using 1.5 mL of Matrigel solution (see Recipe 1); to allow Matrigel to deposit, keep at 37 °C for a minimum of 30 min up to a maximum of overnight.

**Critical:** Always prepare Matrigel-containing solution on ice to avoid premature solidification.

*Notes:*

*1. 1.5 mL of Matrigel solution contains 7.5 μL of pure Matrigel (see Recipe 1). The volume must be adapted by scaling up or down based on the plate format and its surface area.*

*2. We typically store cells in liquid nitrogen at a concentration of 1–2 million cells per cryovial in 1mL of freezing medium. Adjust the number of coated wells accordingly if hiPSC aliquots have a different concentration.*

2. Thaw a hiPSC aliquot from the liquid nitrogen tank.

**Critical:** This protocol was optimized for HMGU1 cells. When using other hiPSC lines, please be aware that the concentration of chemical compounds and the duration of various steps may require adjustment.

3. As soon as the cell aliquot starts to thaw, transfer the contents into 10 mL of prewarmed DMEM/F-12 medium to dilute DMSO contained in the freezing medium.

**Critical:** We occasionally experienced poor formation of the cell pellet with consequent partial cell loss; to promote stable formation of the cell pellet at the bottom of the centrifuged Falcon tube, consider supplementing DMEM/F-12 medium with 0.1% BSA.

4. Centrifuge at 288× *g* for 5 min and remove supernatant.

5. Resuspend in mTeSR1 complete medium (see Recipe 2), supplement with 10 μM ROCK inhibitor, and plate in Matrigel-coated well.

**Caution:** ROCK inhibitor Y-27632 is a chemical substance that requires careful handling. Always refer to the safety information and material safety data sheets (MSDS) for proper precautions when working with this inhibitor.

*Notes:*

*1. In our experience, between 0.5 and 2 million HMGU1 cells can be plated in one well of a 6-well plate at this step. Variations in the actual number of cells per well may arise due to cell mortality shortly after thawing. If using hiPSC lines other than HMGU1, refer to specific protocols to determine the optimal cell seeding density.*

*2. We also used mTeSR Plus medium as an alternative to mTeSR1 medium, with no noticeable differences in hiPSC viability or growth.*

6. Remove the ROCKi-containing medium after 24 h and replace it with fresh mTeSR1 medium without ROCKi.

7. Expand according to standard hiPSC culture protocol.

**Critical:** Requirements of specific hiPSC media and coating protocols can vary depending on the hiPSC lines. If using hiPSC lines different from HMGU1, make sure to adapt these culture steps accordingly. For cell passaging during amplification, we used both Accutase and Versene, obtaining similar results; please refer to the manufacturers' specific protocols and adapt them to the specific requirements of the hiPSC line in use.

**B. hiPSC seeding for neural induction protocol**

1. Prepare the coating of a suitable plate using Matrigel solution (see Recipe 1); to allow Matrigel to deposit, keep at 37 °C for a minimum of 30 min up to a maximum of overnight.

**Critical:** Always prepare Matrigel-containing solution on ice to avoid premature solidification.

*Notes:*

*1. For coating, we use 500 μL of Matrigel solution per well of a 24-well plate, corresponding to 2.5 μL of pure Matrigel. When using other plate or dish types, this volume must be adjusted by scaling up or down based on the plate format and its surface area.*

*2. To reduce the volume of NIM used in subsequent steps, we typically seed cells in smaller plate formats, such as 24-well plates. Adapt the number of wells according to your experimental needs. In our experience, one well of a 24-well plate with confluent hiPSCs can yield approximately 1 million NPs by Day 7 of the protocol.*

2. Wash hiPSCs with prewarmed 1× DPBS to remove dead cells and debris.

3. Incubate hiPSCs with Accutase solution for 5 min at 37 °C; to help detach cells, we normally gently rock the plate at minute 3.

4. Dissociate cells by pipetting up and down several times.

**Critical:** Too harsh pipetting can result in increased cell death; before pipetting too energetically, consider increasing Accutase incubation time to up to 10 min if cells do not detach.

5. Harvest dissociated cells in a 15 mL Falcon tube containing 10 mL of prewarmed DMEM/F-12 to dilute Accutase.

6. After carefully mixing by pipetting up and down, collect 15 μL of cell suspension and load into a Burker chamber for cell counting.

7. Centrifuge cell suspension at 288× *g* for 5 min; discard supernatant.

**Critical:** We occasionally experience poor formation of the cell pellet with consequent partial cell loss; to promote stable formation of the cell pellet at the bottom of the centrifuged Falcon tube, consider supplementing DMEM/F-12 medium with 0.1% BSA.

8. To remove residual Accutase, wash the cell pellet with 5 mL of prewarmed DMEM/F-12 and centrifuge again; discard supernatant.

9. Resuspend the appropriate number of cells in mTeSR1 complete medium supplemented with 10 μM ROCK inhibitor. Remove Matrigel coating medium from the wells to be seeded; seed 50,000 cells per well of a 24-well plate in 500 μL of mTeSR1 medium.

*Notes:*

*1. We also used mTeSR Plus medium as an alternative to mTeSR1 medium, with no noticeable differences in hiPSC viability or growth.*

*2. Scale the number of cells and volume of medium up or down in a proportionate manner based on the seeding surface area when using different plate or dish formats, to maintain a seeding density of 25,000 cells/cm^2^.*

10. Gently rock the plate to ensure cells are uniformly distributed.

11. Incubate at 37 °C with 5% CO_2_.

12. Twenty-four hours after plating, refresh mTeSR1 medium without ROCK inhibitor; the medium volume for one well of a 24-well plate ranges from a minimum of 500 μL to higher volumes (typically 1 mL) if culture medium becomes yellow.

*Note: Scale the volume of medium up or down in a proportionate manner if using different plate or dish formats.*

**Figure 1.**
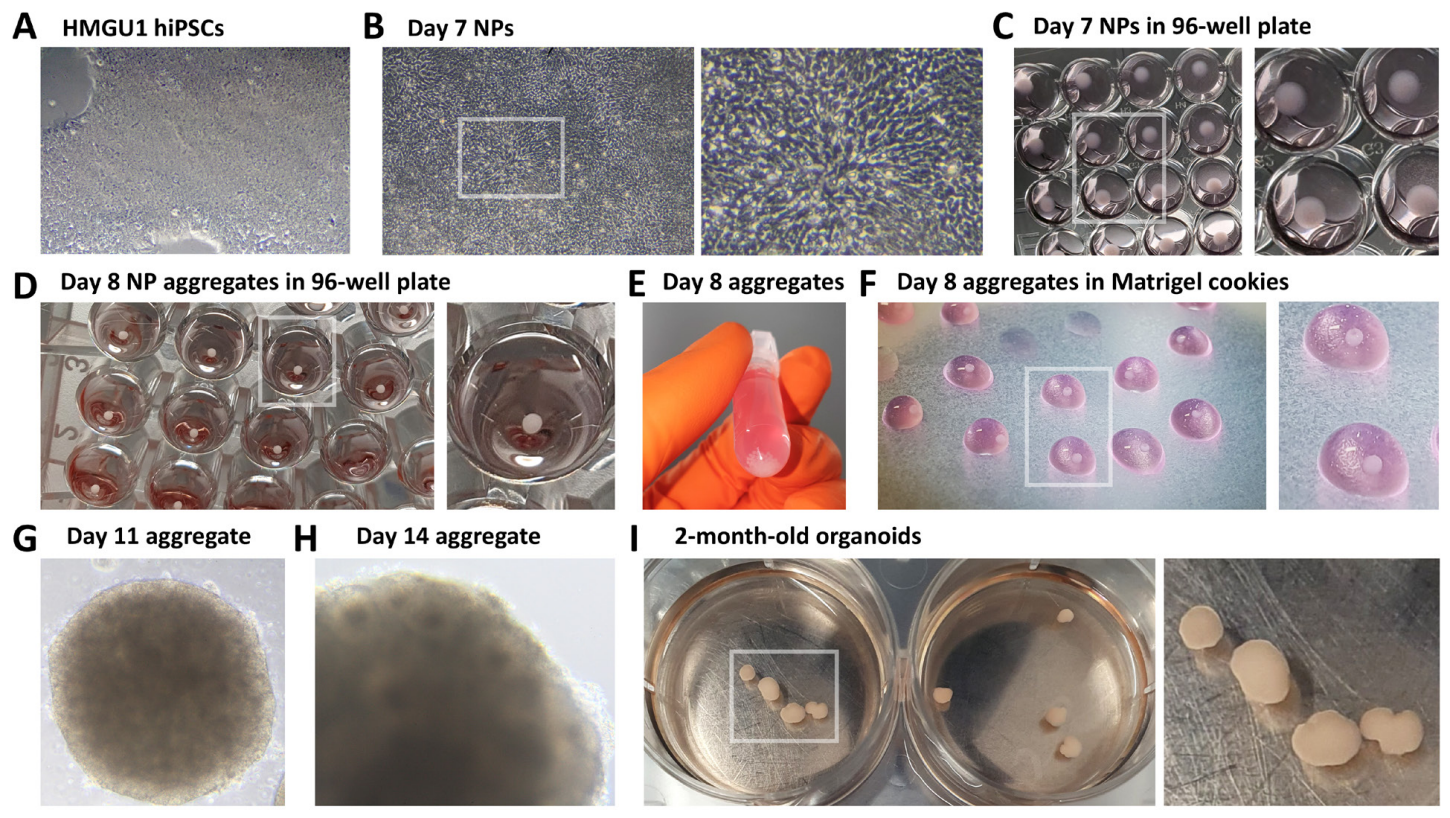
Brightfield images of hybrid 2D/3D protocol for the generation of telencephalic human organoids. (A) Undifferentiated 90% confluent HMGU1 hiPSCs. (B) Day 7 neural progenitors (NPs); a high-magnification inset highlights the elongated, radial morphology of NP cells. (C) Dissociated NPs on Day 7, spun down at the bottom of the wells of a low-adhesive 96-well plate. (D) Spontaneously reaggregated NPs 24 h after dissociation, forming aggregates by Day 8. (E) NP aggregates collected in an Eppendorf tube prior to embedding in Matrigel. (F) Matrigel droplets containing aggregates (referred to as “cookies”) on Parafilm during the inclusion procedure at Day 8. (G) Day 11 NP aggregate embedded in Matrigel. (H) Aggregate at Day 14, with some rosettes becoming visible in brightfield microscopy, indicating NP organization into 3D epithelial structures. (I) Day 60 organoids, shown here in wells of a 6-well plate.

13. Check on hiPSC growth and change medium daily.

14. Monitor cell growth until hiPSCs reach 90% confluency (Figure 1A).

**Critical:** High cell confluency at the start of the neural induction phase is critical for optimal yield of neural progenitors. Lower confluency may result in poor neural induction.

**C. Neural induction on 2D coated surface**

1. Once the hiPSCs have reached 90% confluency, this can be considered the starting point of neural induction (Day 0).

2. Remove mTeSR1 medium.

3. Wash cells with 500 μL of prewarmed DMEM/F-12 medium to remove residual stem cell medium.

4. Add 500 μL of NIM (see Recipe 3 for NIM preparation and Recipe 4 for the addition of chemical inhibitors) and incubate at 37 °C with 5% CO_2_.

**Caution:** NIM medium with chemical inhibitors contains active chemical substances. Always refer to safety information and material safety data sheets (MSDS) for proper handling and precautions when working with these inhibitors.

*Note: As an alternative to XAV-939, we have also successfully tested 3 μM IWR-1-endo as a WNT inhibitor in NIM (see Recipe 4). Both compounds were found to be functionally equivalent in our experiments.*

5. Change NIM daily until Day 7.

**Critical:** If the medium turns yellow in less than 24 h, progressively increase the volume per well up to 1 mL/well.

**Critical:** If cells show detachment or signs of toxicity, reinforce surface coating by dissolving 1 μL of Matrigel per 1 mL of NIM before performing the daily medium change.

**D. 2D-to-3D switch by cell dissociation and reaggregation**

1. At Day 7, hiPSCs should have acquired the morphology of radially oriented neural progenitors, forming either small, radially organized clusters known as neural rosettes or larger, continuous layers of progenitors across the plastic surface (Figure 1B).

*Notes:*

*1. One additional day of culture can be waited before dissociation if cells are still not showing the morphology of neural progenitors.*

*2. At high cell density, it may be difficult to clearly distinguish the typically elongated morphology of neural progenitors and their organization into neural rosettes or larger clusters. Observing cells at high magnification or along the edges of large cellular clusters can be helpful. Refer to Figure 1 for a high-magnification image, and to the original article for additional examples [1].*

2. Wash cells with 1 mL of prewarmed 1× DPBS to remove dead cells and debris.

3. Add 500 μL of prewarmed Accutase solution and incubate for 7 min at 37 °C; to help detach cells, we gently rock the plate after the first 4 min of incubation.

**Critical:** Increase the incubation time up to a maximum of 15 min if cells are not detaching from the surface.

4. Dissociate cells by pipetting up and down several times.

**Critical:** Too harsh pipetting can result in increased cell death; consider increasing Accutase incubation time **before** pipetting too energetically.

5. Harvest dissociated cells in a 15 mL Falcon tube containing 10 mL of prewarmed DMEM/F-12 to dilute Accutase.

6. Continue cell dissociation by pipetting up and down several times, then collect 15 μL of cell suspension and load into a Burker chamber for cell counting.

7. Centrifuge cell suspension at 288× *g* for 5 min; discard supernatant.

**Critical:** We occasionally experience poor formation of the cell pellet with consequent partial cell loss; to promote stable formation of the cell pellet at the bottom of the centrifuged Falcon tube, consider supplementing DMEM/F-12 medium with 0.1% BSA.

8. To remove residual Accutase, wash the cell pellet with 5 mL of prewarmed DMEM/F-12 and centrifuge again; discard supernatant.

9. Resuspend the cells in NIM (see Recipe 4) supplemented with 10 μM ROCK inhibitor; seed 50 μL of NIM containing 60,000 cells per well in a 96-well U-bottom low-attachment plate.

**Caution:** ROCK inhibitor Y-27632, SB-431542, LDN-193189, and XAV-939 are active chemical substances that require careful handling. Always refer to safety information and material safety data sheets (MSDS) for proper precautions when working with chemical inhibitors.

*Notes:*

*1. Adapt the volume of NIM + ROCKi depending on how many cells you counted and how many aggregates you plan to obtain.*

*2. We previously varied this step of the protocol with no noticeable effect by changing the number of cells per well, ranging from a minimum of 50,000 to a maximum of 120,000 cells per well of a 96-well plate.*

**Critical:** Neural progenitors fail to survive dissociation in the absence of ROCK inhibitor.

**Critical:** The 96-well must be a low attachment surface to ensure cells will reaggregate at the bottom without sticking to the plastic surface; also, ensure that Matrigel has not been dissolved into NIM to avoid any coating effect that would promote cell adhesion.

10. Centrifuge the 96-well plate at 200× *g* for 1 min to spin cells down to the bottom of the wells and promote their aggregation (Figure 1C).

11. Incubate overnight at 37 °C with 5% CO_2_.

**E. Embedding of 3D neural progenitor aggregates in Matrigel cookies**

1. Before starting this step, prepare an ice bucket and thaw the volume of Matrigel needed; we normally use 150 μL of Matrigel for 30 aggregates.

2. Prepare P100 Petri dishes containing pieces of Parafilm; spray Parafilm with 70% ethanol solution and let them dry under the hood before placing them inside Petri dishes.

3. Neural progenitors seeded into 96-well low-attachment plates the day before should now have formed spherical aggregates of neural progenitor cells (Figure 1D). Collect the aggregates from the 96-well plate into a 2 mL Eppendorf tube (Figure 1E).

**Critical:** To avoid damaging the aggregates, open the pipette tip used for harvesting by cutting it at a 45° angle with ethanol-sterilized scissors. This angled cut allows safe aspiration of the aggregates, even when the tip touches the bottom of the well.

4. Remove as much NIM as possible from the Eppendorf, leaving the aggregates at the bottom.

**Critical:** Do not completely dry the NP aggregates; always leave a film of liquid covering them.

5. Add Matrigel (150 μL of Matrigel every 30 aggregates) and mix well by gently rocking the Eppendorf.

**Critical:** Matrigel solidifies quickly at room temperature; keep the Eppendorf containing the aggregates on ice as much as possible after adding Matrigel and proceed quickly to the next steps.

6. Cut a 200μL pipette tip using ethanol-sterilized scissors. Remove the Matrigel/aggregates solution from ice, quickly mix by pipetting up and down several times, and then immediately distribute it in small droplets on the Parafilm (Figure 1F).

*Note: Matrigel droplets may vary slightly in size; to ensure reproducibility, consider setting your pipette to a fixed volume (typically 10μL per droplet). However, we did not observe significant differences in neural induction efficiency or organoid growth when small variations in droplet size occurred.*

**Critical:** Ensure at least one aggregate per Matrigel droplet; if not, you can move and redistribute neural progenitor aggregates from one droplet to another using a cut-open P20 tip, as long as the Matrigel has not solidified yet.

7. Close the Petri dish lid and transfer to the incubator for Matrigel solidification (20–25 min required).

8. Prepare a 6-well plate (if using an orbital shaker) or a 12-well plate (if using a SpinΩ mini-bioreactor) with the desired number of wells, each containing 2.5 mL of prewarmed NIM.

9. After 20–25 min at 37 °C, the Matrigel should have solidified; detach Matrigel droplets containing neural progenitor aggregates (named “cookies”) from the Parafilm pieces. To do this, open a P1000 tip with clean scissors and pipette some NIM solution onto the cookies. Gently pipette up and down until the cookies detach from the Parafilm and transfer them into a 6-well or 12-well plate.

**Critical:** Too gentle pipetting will fail to detach the cookies from Parafilm, while too harsh pipetting will break them. Perform a technical try on an empty Matrigel droplet when doing this for the first time to familiarize yourself with the pipetting procedure.

*Note: If using a SpinΩ mini-bioreactor, transfer cookies into a 12-well plate. If using an orbital shaker, use a 6-well plate, as this format allows a better mixing of the medium.*

10. If using SpinΩ mini-rotor, take a clean spinning cover and place it on the top of the 12-well plate containing cookies in NIM medium. Then, plug the cover into the power supply to activate mixing.

*Notes:*

*1. Non-experienced users should refer to the original paper by Qian et al. [2] for additional images and diagrams illustrating the structure of a SpinΩ mini-bioreactor cover and how to properly place it over a 12-well plate, ensuring it covers and closes the plate when in function.*

*2. If 3D-printed with autoclavable plastic, SpinΩ covers can be sterilized in an autoclave. However, in our experience, repeated sterilization cycles can cause partial damage to the plastic. As an alternative, we cleaned SpinΩ covers by soaking them in 70% ethanol for 2 h, then drying them under the cell culture hood and storing them in clean plastic bags.*

**Critical:** Set the rotation of SpinΩ helices at low speed to avoid breaking Matrigel cookies.

**Critical:** Exceeding 2.5 mL of medium per well can result in spillage when placing the cover, increasing the risk of contamination. Ensure no more than 2.5mL of medium per well is added.

11. If using an orbital shaker instead of a SpinΩ mini-rotor, place the 6-well plate on the shaker (speed: 120 rpm).

12. Incubate at 37 °C with 5% CO_2_.

**F. 3D brain organoid culture**

1. At Day 10, prepare fresh NIM medium containing WNT inhibitor (XAV-939 or IWR-1-endo) but without SB-431542 and LDN-193189. Remove as much medium as possible from the 6-well/12-well plate and add 2.5 mL of the new medium.

*Notes:*

*1. If using a SpinΩ mini-rotor, a safe way to remove the spinning cover during media changes is to use an empty 12-well plate as a rack to hold the cover. Use the lid of the empty plate to close the plate containing the cookies when taking it out of the hood (e.g., to observe under a microscope).*

*2. Avoid aspirating cookies when removing the medium. To prevent damaging them, consider using a cut-open P1000 tip. If a cookie is accidentally aspirated, gently return it to the well.*

2. From Day 11 to Day 20 (Figure 1G, H), refresh the medium every 3–4 days by performing a partial change (remove 1.5 mL and replace with 1.5 mL of fresh medium).

**Critical:** If the medium becomes too yellow, indicating high nutrient consumption and acidification, split the cookies into additional wells to reduce the organoid-to-medium ratio.

*Notes:*

*1. Avoid aspirating cookies when removing the medium. To prevent damaging them, consider using a cut-open P1000 tip. If a cookie is accidentally aspirated, gently return it to the well.*

*2. Do not exceed 2.5 mL of total medium volume in SpinΩ plates to avoid spills and contamination risk.*

3. From Day 20 to Day 35, gradually replace NIM with NDM (see Recipe 5) to promote neural differentiation (conversion of neural progenitors into neurons).

*Note: Refresh the medium every 3–4 days.*

**Critical:** If medium becomes too yellow, split cookies into more wells or increase medium change frequency.

4. Starting from Day 35, gradually replace NDM medium with LTSM (see Recipe 6) to promote long-term neuronal survival and continued organoid growth.

*Notes:*

*1. Organoids may increase in size, potentially developing necrotic cores due to limited oxygen and nutrient diffusion. Dissect larger organoids into smaller pieces using ethanol-sterilized forceps to improve survival.*

*2. Detached cells may colonize the plastic surface, contributing to medium consumption. Regularly inspect the wells and, if necessary, transfer organoids to new 6-well (orbital shaker) or 12-well (SpinΩ) plates.*

**Critical:** Always use a cut-open pipette tip when transferring organoids to avoid damaging them.

5. Partially refresh culture medium every 3–4 days during the subsequent months of culture (Figure 1I). Using this protocol, we successfully cultured organoids for up to 8 months.

**G. FGF8-induced increase in regional cell diversity**

1. The regional identity of neural progenitors and neurons can be modulated using morphogens. For FGF8, add 100 ng/mL recombinant FGF8 from Day 12 to Day 50, following the same medium change schedule as in previous sections, with the only change being the addition of FGF8 to NIM, NDM, and LTSM.

**Critical:** Each morphogen has specific concentration and time window requirements. Additionally, variability between hiPSC lines can affect responsiveness. Always adapt dosage and treatment windows based on the specific morphogen used. Testing multiple concentrations and time points is strongly recommended.

## Data analysis

**Figure 2.**
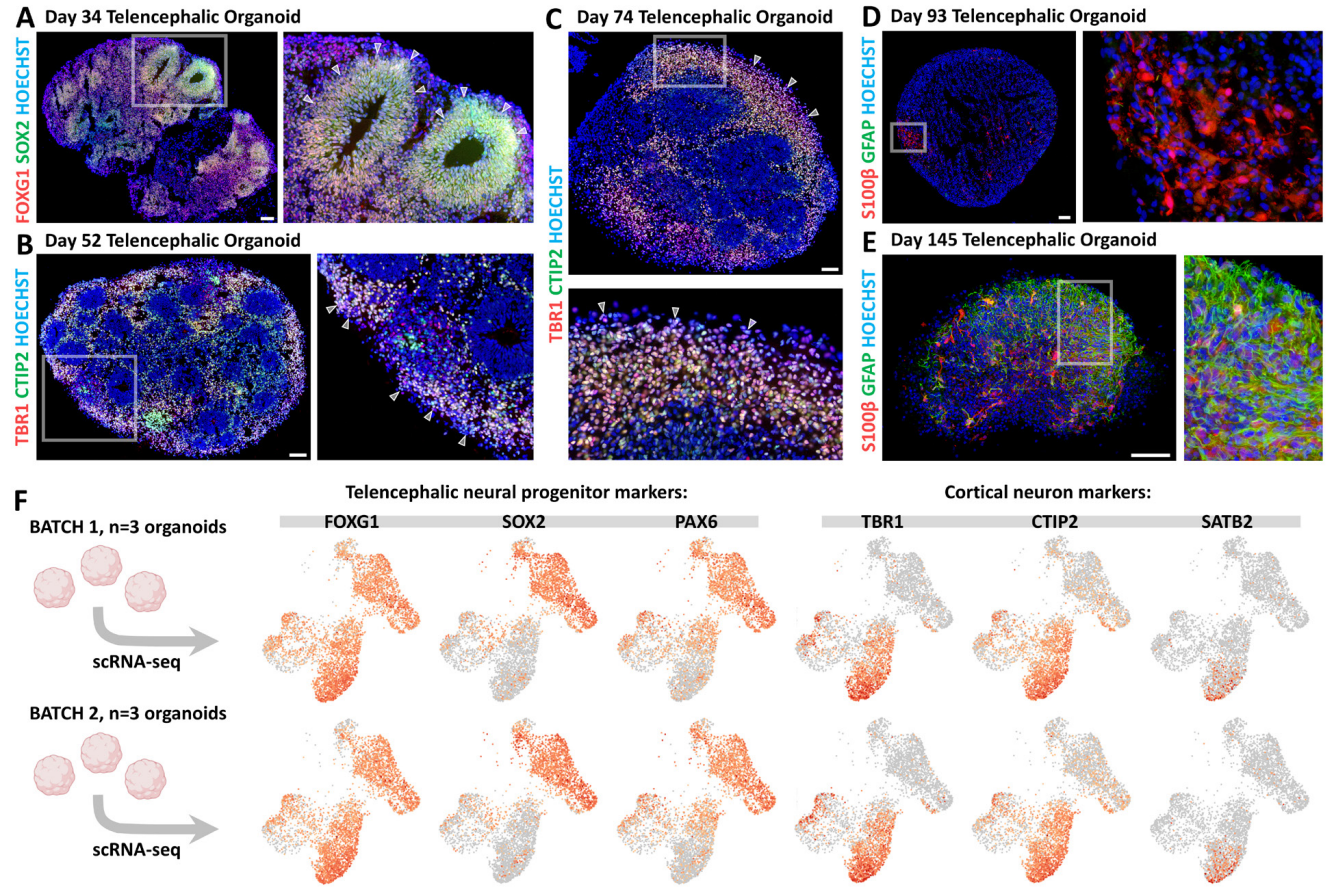
Telencephalic cell type characterization by immunostaining on organoid cryostat sections and single-cell RNA sequencing (scRNA-seq). (A) FOXG1 (red) and SOX2 (green) immunostaining in Day 34 telencephalic organoid. White arrowheads in the high-magnification image indicate the borders of neural progenitor (NP) neural rosettes. (B) TBR1 (red) and CTIP2 (green) immunostaining on Day 52 organoids. High-magnification image highlights TBR1+ CTIP2+ differentiating cortical neurons (white arrowheads). (C) TBR1+ CTIP2+ cortical neurons accumulating on the external layer of a Day 74 organoid. (D, E) S100β (red) and GFAP (green) immunostaining of a Day 93 (D) and a Day 145 (E) organoid, showing the gradual appearance of S100β+ astrocytic progenitors and GFAP+ differentiated astrocytes in telencephalic organoids upon longer culture periods. Scale bars: 100 μm. (F) scRNA-seq data from two independent batches of Day 69 telencephalic organoids (batch 1: top row; batch 2: bottom row). Each batch included three organoids that were pooled prior to dissociation and processing. Expression levels of established markers for various cell types—including telencephalic neural progenitors (FOXG1, SOX2, and PAX6) and cortical neurons (TBR1, CTIP2, and SATB2)—are displayed for both batches. A similar number of marker-positive cells and consistent clustering patterns across batches demonstrate experimental reproducibility. For details on scRNA-seq methods and additional data, please refer to the original article [16].

This protocol results in the formation of FOXG1+ SOX2+ telencephalic neural rosettes or neuroepithelia (Figure 2A), which are surrounded by TBR1+ CTIP2+ cortical neurons (Figure 2B, C). At later stages, S100β+ astrocytic precursors and GFAP+ astrocytes also emerge (Figure 2D, E). Microscope images shown in Figure 2 were obtained by immunostaining of organoid cryosections. The procedures for organoid fixation, sectioning, and immunostaining have been described in detail elsewhere [16,17]. Images were acquired using a Zeiss Apotome microscope and processed with AxioVision software.

## Validation of protocol

Organoid protocols can exhibit significant variability both within and between batches. To validate the robustness of our approach, we took advantage of single-cell RNA sequencing (scRNA-seq) data that we obtained using this protocol [16]. Two independent batches of Day 69 telencephalic organoids were analyzed, each consisting of a pool of three organoids (six organoids in total, derived from two separate culture plates) (Figure 2F). UMAP projections showing the expression levels of telencephalic neural progenitor markers (SOX2, PAX6, and FOXG1) and cortical neuron markers (TBR1, CTIP2, and SATB2) revealed a highly similar number of marker-positive cells and consistent distribution across clusters between the two batches (Figure 2F). These results indicate a reproducible induction of telencephalic cell types at both cellular and molecular levels. For details on the scRNA-seq methodology and additional sequencing data, please refer to the original research article in which this protocol was used and validated:

Bertacchi et al. [16]. FGF8-mediated gene regulation affects regional identity in human cerebral organoids. *eLife* 13: e98096. https://doi.org/10.7554/eLife.98096.

## General notes and troubleshooting

**General notes**

1. As an alternative to XAV-939, we successfully employed IWR-1-endo for WNT inhibition and neural progenitor anteriorization. Each chemical compound must be tested at varying concentrations, as sensitivity and toxicity may differ between hiPSC lines.

2. As an alternative to the 12-well-adapted SpinΩ mini-rotors, orbital shakers can be used to ensure optimal oxygenation and nutrient distribution. We successfully used a CO_2_-resistant Fisher Scientific model (see Equipment), set at 120 rpm. Organoids can be cultured in 6-well plates with 3–4 mL of medium per well to maintain efficient shaking. Notably, other orbital shaker models tested yielded suboptimal results due to uncontrolled increases in incubator temperature.

3. The regional identity of neural progenitors and neurons in organoids can be modulated using morphogens [2,18,19], as we and others recently did with FGF8 [16,20,21]. This protocol may be used to investigate the effects of additional morphogens on regional specification. However, the timing and dosage of treatments with other morphogens should be carefully optimized to ensure maximal efficacy.

**Troubleshooting**

**Problem 1: Poor attachment or viability after thawing hiPSCs.**

Possible cause: Incomplete or ineffective Matrigel coating or insufficient ROCK inhibitor supplementation.

Solution: Ensure Matrigel is fresh, prepared on ice, and properly deposited at 37 °C for 30 min to overnight. Always supplement the medium with ROCK inhibitor after thawing to prevent hiPSC cell death.

**Problem 2: Poor neural induction.**

Possible cause: Degradation or oxidation of chemical drugs used in induction (e.g., SB-431542, LDN-193189, XAV-939).

Solution: Always store chemical drugs at -20 °C, protected from light. For long-term storage, store at -80 °C. If poor induction is observed, prepare fresh aliquots of the chemical drugs and use the medium supplemented with them in the following days.

**Problem 3: Heterogeneous or poor neural rosette formation.**

Possible cause: Low confluency at the start of neural induction or suboptimal culture conditions.

Solution: Ensure that hiPSCs reach at least 90% confluency before initiating neural induction, as insufficient confluency on Day 0 can lead to reduced neural progenitor yield. Maintain proper culture conditions by changing the medium daily and increasing its volume if it turns yellow within 24 h.

**Problem 4: High cell mortality after NP dissociation at Day 7.**

Possible cause: No ROCK inhibitor was added.

Solution: Always add ROCK inhibitor to prevent cell death when dissociating NPs. NPs can also be pre-treated with ROCKi for 2 h prior to dissociation.

**Problem 5: Poor aggregation of neural progenitors after 2D-to-3D switch.**

Possible cause: Incorrect or insufficient cell seeding density; no ROCK inhibitor added; excessive mechanical stress during pipetting leading to cell death.

Solution: Ensure that the correct number of cells (60,000 per well in a 96-well low-attachment plate) is seeded, and always include 10 μM ROCK inhibitor. Too few cells or a lack of ROCK inhibitor can impair survival and aggregation.

**Problem 6: Yellowing of medium in 3D organoid culture.**

Possible cause: High nutrient consumption and acidification of the medium, which could affect organoid growth.

Solution: Split organoids into more wells to reduce the organoid-to-medium ratio if yellowing occurs. If using a SpinΩ mini-rotor or orbital shaker, ensure medium volume does not exceed recommended amounts to avoid spillage and contamination.

**Problem 7: Ineffective regionalization or poor response to morphogens.**

Possible cause: Incorrect concentration or timing of morphogen administration; variability between hiPSC lines.

Solution: Optimize the concentration of morphogens and ensure they are added to NIM, NDM, and/or LTSM media at the correct time. Test different morphogen concentrations and time points based on the hiPSC line to achieve the best results.
